# Development and Validity Evidence of the Retirement Decision Inventory (RDI) in Brazil and Portugal

**DOI:** 10.1007/s10823-026-09603-x

**Published:** 2026-10-28

**Authors:** Lucia Helena Freitas Pinho França, Larissa Maria Gabardo-Martins, José Joaquim Marques da Costa, Margarida Pedroso de Lima, Luiza Contage, Helena Maria Almeida Macedo Loureiro

**Affiliations:** 1 Laboratório de, Envelhecimento No Contexto Organizacional E Social, Programa de Pós-Graduação Em Psicologia, Universidade Salgado de Oliveira, Rio de Janeiro, Brazil; 2 Faculdade de Psicologia E de Ciências da Educação, Universidade de Coimbra, Coimbra, Portugal; 3 Escola Superior de Saúde, Universidade de Aveiro, Aveiro, Portugal

**Keywords:** Retirement, Judgment and decision-making, Scale development/measurement/validation, Cross-cultural issues

## Abstract

Retirement decision-making is a complex process influenced by cognitive, social, and emotional factors. Although retirement decision-making has been widely examined, there is a lack of specific instruments designed to assess older workers’ intentions. This study aimed to construct and validate the Retirement Decision Inventory (RDI), designed to assess four dimensions: early retirement, full retirement, bridge employment (transitional work post-retirement), and late retirement. An exploratory factor analysis (EFA) on a Brazilian sample (*n* = 764) reduced an initial 20-item pool to 13 items. A subsequent confirmatory factor analysis (CFA) on an independent Portuguese sample (*n* = 607) confirmed the four-factor structure, showing acceptable fit (e.g., CFI = .969, RMSEA = .097). Internal consistency was satisfactory (α ranging from .76 to .87). Multigroup CFA supported the configural and metric equivalence of the model across cultures. Mean comparison analyses revealed cross-cultural differences: Portuguese participants reported stronger tendencies toward full retirement, whereas Brazilian participants showed greater inclination toward continued labor market participation. The RDI is a reliable and structurally valid instrument with satisfactory psychometric properties for cross-cultural assessment of retirement intentions. It offers a valuable tool for retirement planning initiatives and can inform organizational and public policies aimed at promoting active aging and retaining older workers.

## Introduction

Retirement decision-making is a multifaceted process that involves various cognitive, emotional, physical, financial, social, and situational factors. Although decision-making has been the subject of interest from various disciplines, psychology has made a significant contribution to its understanding and application. Decision-making research in psychology is largely grounded in Ward Edwards'' (1954) subjective utility theory, according to which people make decisions based on expected consequences, considering the usefulness or value of their choices and possible outcomes or consequences (Edwards, 1954).

Psychology can offer a more comprehensive and realistic approach to the phenomenon of decision-making (Edwards, 1954). This decision encompasses three elements: (i) selecting an option from among several possibilities; (ii) carefully evaluating each alternative beyond a simple selection; and (iii) deciding based on an objective or goal (Lunenburg, 2010).

Applied to the retirement context, decision-making theories elucidate how older workers evaluate multiple, often competing alternatives under conditions of uncertainty, long-term consequences, and institutional constraints. Retirement decisions rarely involve a binary choice between working or not working; instead, they reflect a complex process in which individuals weigh continuity versus disengagement from work, anticipated gains and losses, identity considerations, and expectations regarding aging and future well-being (Feldman & Beehr, 2011; Wang et al., 2008). From this perspective, retirement intentions can be understood as psychologically grounded decision outcomes rather than isolated or purely structural events.

In the behavioral sciences, retirement is a transition that involves attitudes such as the initial perception of the event, which can lead to planning, enabling older workers to make adaptive and well-being-oriented decisions (Amani & Fussy, 2023; Blinded for review, 2022; Henkens et al., 2021). This transition considers aspects related to job satisfaction (Tambellini, 2023), commitment to work (Henkens & van Solinge, 2021), identity (Crary et al., 2025), the perception of gains and losses in the face of retirement (França & Vaughan, 2008), and expectations in the face of a new stage of life (Feldman & Beehr, 2011). Consequently, planning should be encouraged so that older workers can make their decisions with greater confidence (Liu et al., 2022).

Although many studies examine predictors of retirement-related decisions, relatively few focus on the decision itself – whether to continue working or leave the labor market (Amorim et al., 2022). To address this gap, we developed a new instrument to assess retirement-related decisions, described in the Method section.

From a gerontological perspective, retirement decisions reflect different configurations of engagement and disengagement with work in later life. Drawing on continuity theory and disengagement theory (Atchley, 1989; Cumming & Henry, 1961), these decisions can be conceptualized according to the degree and form of continuity individuals seek to maintain in their working lives. Early retirement and full retirement represent forms of disengagement, differing primarily in timing and institutional alignment, whereas bridge employment and late retirement reflect continuity-oriented decisions, characterized by partial or full maintenance of work roles. The retirement decision can involve four main options: (i) early retirement (ER), (ii) full retirement (FR), (iii) bridge employment (BE), and (iv) late retirement (LR) (Amorim et al., 2022; Dingemans & Henkens, 2019; Feldman, 1994; França & Carneiro, 2013; Wang et al., 2008). Thus, these four options represent qualitatively distinct decision pathways rather than merely empirical categories.

Early retirement is defined by Feldman (1994) as the forced withdrawal from work before a specific regulatory age. However, early retirement may also occur when older workers have sufficient financial resources to exit the workforce before the legal age. Retiring at the statutory age entails accessing pension benefits and other secondary sources of income. In Brazil, this possibility occurs when the worker reaches the age (62 for women, 65 for men) and the minimum contribution time (15 years) (Instituto Brasileiro de Geografia e Estatística, 2023). In Portugal, it occurs at 66 years and 9 months or after 40 years of contribution (Portugal, 2025).

Bridge employment involves leaving one''s main job due to retirement to dedicate oneself to another job with flexible working hours (França & Carneiro, 2013). According to Kim and Feldman (2000), bridge employment can take place before the worker definitively leaves the workforce, involving a combination of reduced working hours, less stress or responsibility, greater flexibility, and less physical demand. It can also occur when the worker needs some financial supplement for when they stop working altogether. Common examples include entrepreneurs, freelancers, or service providers (Beehr & Bennett, 2015; Mizuochi, 2024).

Although bridge employment is rare in many countries, research on the employability of older workers who seek bridge employment is also limited. It is important to note that bridge employment is neither a precarious nor an involuntary job (Alcover & Londoño, 2021; Londoño-Moreno et al., 2023). Rather, it can be a beneficial option for older workers, organizations, and policymakers, given labor market shortages and the need to accommodate employable older workers who wish to continue working.

Finally, late retirement occurs when older workers, even after being entitled to a retirement pension, remain in the same job or another with the same working hours, organization, and working conditions (Axelrad, 2018; Beehr & Bennett, 2015). This option can allow them to postpone retirement and receive more advantageous financial benefits. Although it allows them to accumulate more financial resources to ensure greater well-being in retirement, it is an option that is conditional on the worker''s physical and cognitive conditions (Li et al., 2021). However, the option to continue working is not a free choice, considering that many workers, especially Brazilians, have not had the opportunity to financially plan their retirement and/or pensions are insufficient to cover basic living expenses (França & Hershey, 2018). Although physical (health), cognitive, and financial data are crucial aspects when analyzing the reasons why older workers decide to retire, the scope of this study was not to identify or compare the predictors of the retirement decision. Therefore, the following proposal is based on the construction of an inventory to be tested for the first time in Brazil and Portugal, countries sharing historical and cultural ties yet differing significantly in retirement systems, demographics, and climate.

From a gerontological and life-course perspective, retirement decision-making is embedded in cultural, institutional, and labor-market contexts. Active aging and life-course frameworks emphasize that decisions in later life are shaped not only by individual preferences but also by welfare regimes, pension systems, and normative expectations regarding aging and productivity (Henkens & van Solinge, 2021; van der Horst & Vickerstaff, 2022). Cross-cultural research is therefore essential to distinguish theoretically robust decision dimensions from context-specific expressions of retirement.

Beyond institutional and demographic differences, retirement decision-making is culturally embedded. Cross-cultural research in gerontology highlights that retirement is not a universal, purely economic transition, but a social construct whose meaning, timing, and experience vary across cultural contexts. Luborsky and LeBlanc (2003) demonstrate that retirement has historically taken diverse forms across societies and that Western models of retirement may not capture culturally distinct practices and expectations in non-Western settings. This analytic perspective underscores that retirement must be understood within culturally specific frameworks of aging, work, and social roles, not merely as an institutional milestone.

Moreover, recent cross-cultural gerontological research suggests that the meanings ascribed to life after retirement are highly influenced by culturally specific values and social practices. Kamath and Dsouza (2025) found that in a collective cultural context, older adults interpret leisure and productive activities after formal retirement not merely as individual choices but as practices shaped by family roles, intergenerational expectations, and culturally embedded social obligations, demonstrating that retirement experiences extend beyond economic disengagement to reflect culturally normative orientations toward aging and social participation.

In Portugal, the proportion of retirees has increased significantly, often without adequate preparation for the transition. Many retirees end up taking on other roles, such as caring for their grandchildren or working in the rural sector (França et al., 2022; Instituto Nacional de Estatística, 2020). This scenario contrasts with Brazil, where continuing to work after retirement is often a necessity, not a choice (Andrade & Torres, 2020; França et al., 2013; França et al., 2022).

Retirement experiences may nevertheless differ across Brazil and Portugal because retirement decisions are embedded in distinct socioeconomic and institutional contexts (United Nations Development Programme [UNDP], 2024). In Portugal, retirement has been described as a period associated with greater peace, leisure, and family involvement, including support for descendants (Fonseca, 2012; Loureiro et al., 2022). In Brazil, retirement is more heterogeneous and, for many older adults, continued employment may remain important for financial security and family support (Andrade & Torres, 2020; Escorsim, 2021; França et al., 2022; Moura et al., 2023). These differences provide a relevant context for examining whether retirement intentions are expressed similarly across the two countries.

Concerning the employability of older workers, many Brazilians wish to continue working either through bridge employment or by continuing to work full-time (França & Carneiro, 2013). However, bridge employment is not institutionalized in either Brazil or Portugal (Lowden et al., 2021), although the practice occurs mainly in the informal market in Brazil (Henkens et al., 2021).

Importantly, the present study does not aim to compare pension systems, socioeconomic development, health conditions, or public policies between Brazil and Portugal. Rather, these contextual differences provide the rationale for testing whether the underlying dimensions of retirement decision-making can be meaningfully assessed across national contexts. Establishing measurement invariance is therefore essential before interpreting cross-national differences in retirement intentions. What this study examines is the retirement decision itself and the desires and intentions underlying workers’ choices, considered as a dependent variable from a multilevel perspective: individual/micro (workers’ planning of continued labor-market participation or retirement) (van der Horst & Vickerstaff, 2022); organizational/meso (age-diversity management policies and practices) (Seidl et al., 2022; van der Horst & Vickerstaff, 2022); and governmental/macro (policies addressing pension-system sustainability while maintaining older workers’ employability amid labor shortages) (Henkens & van Solinge, 2021; República Portuguesa, Ministério do Trabalho, Solidariedade e Segurança Social, 2024). Therefore, given the cultural similarities and contextual differences between Brazil and Portugal, it is appropriate to construct and test the RDI in both countries. This study aimed to present the RDI, its evidence of psychometric validity, and invariance between the two countries. The term “inventory” denotes a multidimensional instrument in which subscales are interpreted independently rather than summed into a global score.

Finally, this cross-cultural study between Brazil and Portugal is part of a broader transcultural research project conducted across four countries, addressing both retirement decision-making (including the assessment instrument and its predictors) and the employability of older workers (França, 2022). Among the manuscripts resulting from this broader project, particular attention should be given to the study examining the RDI in Spain and Colombia (Londoño-Moreno* et al., *2026).

## Method

### Participants

A total of 1,371 workers from Brazil (55.7%) and Portugal (44.3%), aged 50 or over, participated in the study.

### Brazilian Participants

The participants lived in 24 states plus the Federal District, with the Southeastern states being the most represented: Rio de Janeiro (28.1%), São Paulo (18.7%) and Minas Gerais (10.6%). In terms of gender, the majority of respondents were female (67.1%). As for marital status, 67.1% were married or living together; 19.2% were widowed, divorced, or separated; and 13.6% were single. In terms of level of education, a large proportion had Graduate Certificate/Specialization (Lato Sensu) degrees (32.6%), followed by Master''s degrees (18.2%), undergraduate degrees (16.6%), high school degrees (12.2%), PhDs (10.9%), post-doctoral degrees (5.1%) and elementary school degrees (4.5%). The majority of participants self-identified as white (68.1%), followed by mixed-race (Pardo) (22.8%), black (7.1%), and indigenous or asian (2.1%). Age ranged from 50 to 80 years (*M* = 57.6. *SD* = 6.6).

### Portuguese Participants

In Portugal, the sample was primarily recruited from the districts of Coimbra (26.8%), Aveiro (12.7%) and Porto (9.2%). Most of the respondents were female (52.7%). Regarding marital status, 72.2% were married or cohabiting, and 20.8% were widowed, divorced, or separated. In terms of education level, most of the respondents were at level IV (29.7%), basic education (18.5%), and level I (13.7%). The ages of the professionals ranged from 50 to 85 years (*M* = 58.8, *SD* = 6.3).

A comparison of education levels in Brazil and Portugal reveals similar structures. Brazilian Primary education consists of two cycles (5 and 4 years), which corresponds to the three cycles of Basic education in Portugal; both total 9 years of study. Similarly, the three-year High School in Brazil is equivalent to the Secondary Education level (High School equivalent) in Portugal.

When asked to select from four retirement options, 47.2% of respondents indicated they were considering bridge employment, while 27.2% preferred full retirement during the statutory period. Notably, cross-national differences emerged: 38.9% of Portuguese respondents (compared to 17.7% of Brazilian respondents) intended to retire during the legal period, whereas 60.5% of Brazilian respondents (compared to 30.5% of Portuguese respondents) favored bridge employment. With respect to early retirement, 20.6% of Portuguese respondents expressed this preference, compared to only 6.5% of Brazilian respondents, though these percentages remained relatively modest overall. Table 1 presents the complete data regarding retirement decisions.

**Table 1:** Frequencies and percentages of stated retirement preferences by country

Dimension	Brazil	Portugal
Early retirement	N = 50 (6.5%)	N = 125 (20.6%)
Full retirement	N = 135 (17.7%)	N = 236 (38.9%)
Bridge employment	N = 462 (60.5%)	N = 185 (30.5%)
Late retirement	N = 117 (15.3%)	N = 61 (10.0%)
Total	N = 764 (100.0%)	N = 607 (100.0%)

### Instruments

A structured questionnaire was administered to collect data. The first section provided an introduction detailing the research purpose, confidentiality of responses, a thank you message for participants'' cooperation, and instructions for completing the questionnaire. Next, participants completed the Informed Consent Form (ICF) and a sociodemographic questionnaire, which gathered information on gender, marital status, age, place of residence, self-perceived health, ethnicity, education level, employment sector, organization type (public, private, or other), proximity to retirement, family income, dependents, and retirement decision preferences. The questionnaire concluded with the Retirement Decision Inventory (RDI). The instrument was translated from Portuguese into English and backtranslated to verify consistency from Portuguese in Portugal and Brazil and culturally adapted.

### Development of the Retirement Decision Inventory (RDI)

Initial items were adapted from a comprehensive literature review (Amorim et al., 2022), which collected 29 items see Table 2. In this compilation, the intention was grouped as follows: Intention to retire early; Intention to continue working/return to work; Intention to retire completely; Intention to undertake bridge employment.

**Table 2:** Retirement intentions described in the literature (Amorim et al., 2022)

Dimension	Items
Intention to retire early	I would like to retire early if I can
	I would like to stop working as soon as possible
	I hope to retire early in the near future
	I intend to stop working before 65
	I have already considered retiring before reaching the legal retirement age
	I would like to retire early if I can afford it
Intention to continue working/return to work	Postponing retirement is not a problem for me personally
	It''s important for me to continue working after the formal retirement age
	Barring unforeseen circumstances, I would remain with this organization indefinitely
	I would like to work for my current employer in retirement
	I would like to work for another retired employer
	I would like to become self-employed in retirement
	Even when I can retire, I will continue to work
	I believe that if I retire at the age set by the legal criteria, I will feel the urge to return to work
	I don''t see any way of being able to retire in the near future
	Given my conditions, stopping work is not a possibility
Full retirement intention	I don''t see myself continuing to work in my current job for much longer
	I hope to start receiving a pension in the near future
	I intend to retire in the near future
	As soon as I can retire, I''m going to stop all kinds of professional activity
	For me, retirement and work don''t go very well together
	Whatever the nature of the work, I don''t intend to work during retirement
Intention to carry out bridge employment	Working part-time during retirement is not a disadvantage for me
	During retirement, the possibility that I will return to work part-time in my current field of activity is not excluded
	Even when I can retire, I will continue to work, but I will reduce my actual working hours
	Even when I can retire, I will continue working, but changing the type of work
	As soon as I reach the legal criteria for retirement, I intend to become self-employed
	As soon as I meet the legal criteria for retirement, I intend to work in a new job
	I''m interested in taking on a temporary job until I retire for good

The construction of an instrument that can measure the decision intention to retire will contribute to the advancement of the retirement decision construct, as well as to organizations and professionals who want to work with older workers in the career-retirement transition process (Amorim et al., 2022). Accordingly, the RDI seeks to provide a diagnostic framework comprising a set of items organized into dimensions that facilitate reflection by older workers organized into the four dimensions previously identified, facilitating reflection by older workers regarding their retirement options.

Thus, the next stage focused on the redefinition of some items, adding new content and removing existing elements to avoid lengthy sentences, multiple concepts per item, and ambiguous, extreme, negative, or overly technical language.

Thus, initially, the RDI included 20 items arranged in four dimensions. It is important to note that this inventory suggests an order of intentions ranging from the intention to move away from work to total attachment to it. Scores for the four subscales are computed separately, representing a continuum from disengagement (early retirement) to work attachment (late retirement). Items are rated on a Likert scale ranging from 1- strongly disagree to 5- strongly agree. Each dimension is recommended for use as a dependent variable reflecting the employee''s intention to reduce or cease work engagement. Subscale scores are calculated by averaging the items within each dimension.

The research instrument subsequently underwent content analysis and semantic validation. Content analysis involved assessment by 20 expert judges, including researchers and practitioners specializing in organizational behavior, aging, and retirement. Semantic validation ensured item clarity and comprehensibility by identifying flaws or inaccuracies, eliminating ambiguities and leading statements, and verifying appropriate grouping within the designated decision dimensions (see Table 3).

**Table 3:** Retirement decision inventory, initial version

Dimension	Items
Early retirement	RDI1- I would like to retire before the legal retirement period (age + length of service)
	RDI2- I can and will retire early
	RDI3- I would like to stop working as soon as possible
	RDI4- I don''t see myself working to retirement age or length of service
	RDI5- I need to retire before the legal retirement period
Full retirement	RDI6- I''ll retire when I meet the minimum requirements for retirement
	RDI7- When I retire, I won''t work at anything else
	RDI8- For me, retirement and work don''t go very well together
	RDI9- I want to retire and feel free of all work
	RDI10- In retirement, I will devote myself to non-work-related projects
Bridge employment	RDI11- If possible, I would like to continue working part-time in the same company
	RDI12- I would like to have a job in another company, with reduced hours
	RDI13- I would like to work on a different project for a fixed period
	RDI14- After my retirement, I intend to work in other activities a few days a week
	RDI15- I need to continue working, even if it''s a temporary job
Late retirement	RDI16- If I could, I would stay with this company indefinitely
	RDI17- I''m going to retire and work full-time for another company
	RDI18- I''m going to retire and work for myself, full time
	RDI19- I''m going to retire and work something else, but full time
	RDI20- Stopping work is not a possibility for me

### Data Collection Procedures

An online application was used, with participants recruited through social networking platforms such as Facebook, Instagram, LinkedIn, and WhatsApp. In the invitation, they were given brief explanations of the research objectives, followed by a link that took them directly to the survey''s home screen. Data collection took place from August to December 2022.

### Ethical Considerations

The research was, submitted to the Research Ethics Committees in Brazil and Portugal, and was approved under protocol CAEE: 19,575,219.2.0000.5289, in Brazil, and by the Ethics Committee of the Health Sciences Research Unit: Nursing School of Coimbra (UICISA:E), in Portugal, with approval under protocol 880/05–2022. All participants provided informed consent electronically and were informed about the voluntary and anonymous nature of the study.

Once completed, the respondents'' information was kept on file to make up the research database. Only those respondents who agreed to take part in the survey by signing an informed consent form were accepted.

### Data Analysis Procedures

Exploratory and confirmatory factor analyses were used to verify the structure of the instrument. To provide cross-validation using independent samples and to reduce capitalization on chance, the exploratory and confirmatory analyses were conducted in different national samples. This strategy follows recommendations that emphasize the use of independent samples for exploratory and confirmatory purposes, as confirmatory factor analysis conducted after exploratory procedures within the same dataset, even when the sample is randomly split, does not constitute a true test of replication and may yield a false sense of confirmation (Flake et al., 2017). Moreover, splitting a single sample can reduce statistical power and compromise factor stability (Worthington & Whittaker, 2006). Accordingly, the exploratory factor analysis was conducted using the Brazilian sample, whereas the confirmatory factor analysis was performed using the Portuguese sample, allowing for a more rigorous cross-validation of the factor structure and providing additional evidence of construct validity.

The Exploratory Factor Analysis (EFA) was conducted in R (R Core Team, 2021) using the *psych* package (Revelle, 2014). Sampling adequacy was assessed with the Kaiser–Meyer–Olkin (KMO) measure and Bartlett''s test of sphericity. The extraction method was principal axis factoring, with oblique rotation. The factor retention criterion was parallel analysis (Iacobucci et al., 2022).

Confirmatory Factor Analysis (CFA) was conducted using the R *Lavaan* package (Rosseel, 2012). Considering that the Shapiro–Wilk test showed that the sample violated the normality assumption, it was decided to adopt the WLSMV (robust weighted least squares) estimator. The fit indices were assessed according to the recommendations of Xia and Yang (2019): χ^2^/df < 5; CFI > 0.95; TLI > 0.95; RMSEA < 0.08. The instrument''s internal consistency was analyzed using Cronbach''s Alpha coefficient and McDonald''s Omega. Measurement invariance between the Brazilian and Portuguese samples was tested via Multi-Group CFA (MGCFA; Fischer & Karl, 2019). To this end, models were tested in which the number of items and factors (configural invariance), factor loadings (metric invariance), and thresholds and scalars (scalar invariance) were fixed. The differences between the models'' fits were assessed using the chi-squared difference (Δχ^2^) and the CFI (ΔCFI) (Fischer & Karl, 2019).

Finally, the different retirement decision dimensions were computed by averaging their respective items. Subsequently, descriptive statistics (means and standard deviations) were calculated separately for the Brazilian and Portuguese samples. Group differences between Brazil and Portugal were examined using the Mann–Whitney U test for independent samples, given the violation of the normality assumption and the ordinal nature of the response scales. All analyses were conducted in R (R Core Team, 2021) using RStudio.

## Results

### Exploratory Factor Analysis (EFA)

In the EFA, using the Brazilian sample, the results showed that the KMO was equal to.83, demonstrating the adequacy of the sample. Bartlett''s test was significant (*χ*^*2*^ = 5,765.44; *p* <.001), indicating that the correlation matrix Table 1 between the items allowed factor analysis to be carried out. Parallel analysis initially suggested a five-factor solution. However, this solution resulted in several items (6, 10, 20) with low factor loadings (<.40) or problematic cross-loadings. Consequently, the four-factor structure (predicted in the theoretical model developed a priori) was tested. In this four-factor solution with 20 items. Item 6 did not exhibit a primary loading above.40 on any factor and was excluded. Four additional items (11, 15, 16, and 20) showed cross-loadings above.30 on more than one factor and were also excluded. The resulting 15-item, four-factor solution showed all items loading above.40 on a single factor, with no problematic cross-loadings above.30. Table 4 shows the results of the exploratory factor analysis of the latest version, with factor loadings and commonality. After the exclusion of these items, a new exploratory factor analysis was conducted with five factors and 17 items. In this solution, Item 17 also showed salient cross-loadings (≥ 0.30) across multiple factors. However, its exclusion would result in one factor being defined by only two items, which is generally considered inadequate for factor interpretability and stability.

**Table 4:** Results of the exploratory factor analysis (Oblimin Rotation) for the 15-item RDI in the Brazilian sample

Items	Early retirement	Full retirement	Bridge employment	Late retirement	h^2^
RDI1—I would like to retire before the legal retirement period (age + length of service)	.72				.49
RDI2—I can and will retire early	.45				.28
RDI3—I would like to stop working as soon as possible	.51				.40
RDI4—I don''t see myself working to retirement age or length of service	.73				.54
RDI5—I need to retire before the legal retirement period	.69				.59
RDI7—When I retire, I won''t work at anything else		.74			.52
RDI8—For me, retirement and work don''t go very well together		.63			.44
RDI9—I want to retire and feel free from all work		.78			.64
RDI10—In retirement, I will devote myself to non-work-related projects		.54			.30
RDI12—I would like to have a job in another company, with reduced hours			.53		.52
RDI13—I would like to work on a different project for a fixed period of time			.76		.63
RDI14—After my retirement, I intend to work in other activities a few days a week			.81		.66
RDI17—I''m going to retire and work full-time for another company				.81	.72
RDI18—I''m going to retire and work for myself, full-time				.79	.62
RDI19—I''m going to retire and work something else, but full-time				.82	.59

### Confirmatory Factor Analysis (CFA)

**Fig. 1:**
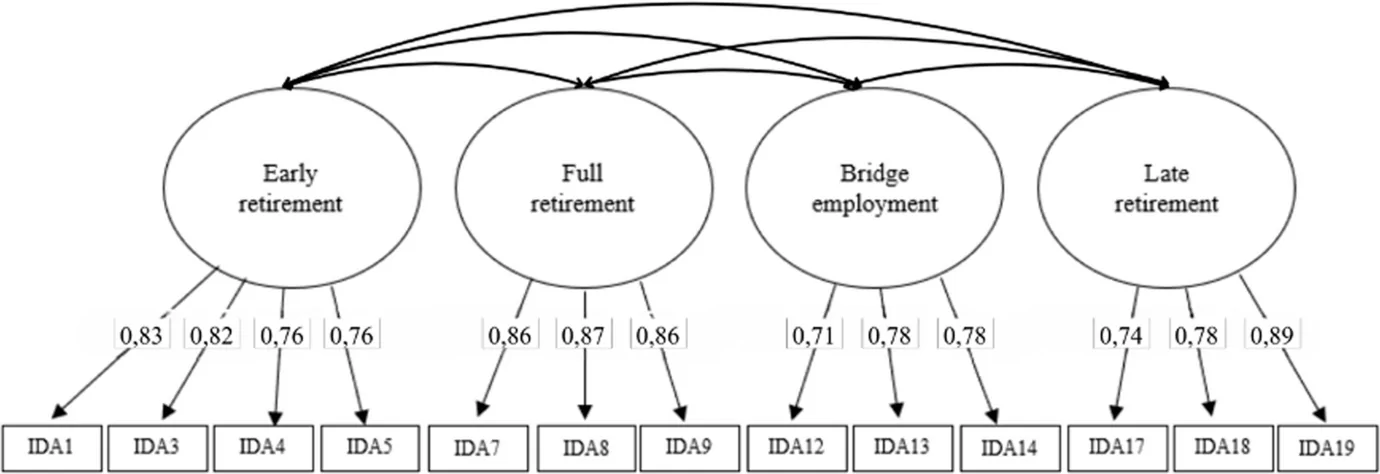
Structure of the RDI after Confirmatory Factor Analysis, in Portugal sample Note. For clarity of presentation, correlations among the latent variables are not depicted in the figure. The estimated latent correlations were: Early retirement–Full retirement =.66; Early retirement–Bridge employment = −.12; Early retirement–Late retirement = −.16; Full retirement–Bridge employment = −.49; Full retirement–Late retirement = −.39; and Bridge employment–Late retirement =.68. All correlations were statistically significant (*p* <.001)

Regarding the CFA for the Portuguese sample, the model was initially tested with the version found in the EFA, i.e. 15 items and four factors, which obtained the following fit indices: χ^2^(df) = 685.27 (84); CFI =.947; TLI =.933; RMSEA =.109 (.101 to.116). However, in this model, two items (2 and 10) had factor loadings below.50 (.44 and.33, respectively). Furthermore, these were the same items that showed low communality in the EFA (.28 and.30, respectively). Therefore, a new model was tested with the exclusion of these items. The structure of 13 items and four factors showed the best-fit indices: χ^2^(df) = 389.57(58); CFI =.969; TLI =.959; RMSEA =.097 (.088 to.106). The final structure of the RDI consisted of 13 items distributed across four first-order factors Fig. 1. The factor loadings of the items in this model ranged from.71 to.89 (M = 0.80). Although the RMSEA remained above conventionally recommended values, this result was interpreted cautiously and in conjunction with the other fit indices. Simulation evidence indicates that the behavior of SEM fit indices depends not only on model misspecification but also on model characteristics, including the number of observed variables. Specifically, Shi et al. (2019) demonstrated a systematic model-size effect on RMSEA, such that models with fewer observed variables tend to yield higher RMSEA values than larger models under comparable levels of model specification. Therefore, rigid reliance on a single RMSEA cutoff may lead to conclusions that differ from those suggested by other indicators of model fit. In the present study, despite the elevated RMSEA, the incremental fit indices showed favorable values (CFI =.969; TLI =.959), and the standardized factor loadings were consistently high (.71–.89; M =.80). Accordingly, model adequacy was evaluated based on the overall pattern of fit indices and parameter estimates rather than on the RMSEA in isolation.

The internal consistency indices were calculated using Cronbach''s Alpha (α) and McDonald''s Omega (Ω). In the total sample, these indices were equal to α =.85 and Ω =.85 (early retirement), α =.86 and Ω =.87 (full retirement), α =.72 and Ω =.74 (bridge employment), and α =.77 and Ω =.79 (late retirement). In the Brazilian sample, these indices were equal to α =.82 and Ω =.79 (early retirement), α =.83 and Ω =.84 (full retirement), α =.70 and Ω =.71 (bridge employment), and α =.76 and Ω =.79 (late retirement). In the Portugal sample, these indices were equal to α =.86 and Ω =.81 (early retirement), α =.87 and Ω =.87 (full retirement), α =.76 and Ω =.76 (bridge employment), and α =.78 and Ω =.81 (late retirement).

### Invariance Analysis

We conducted a Multigroup Confirmatory Factor Analysis (MGCFA) to examine measurement invariance of the RDI structure across the Brazilian and Portuguese groups (Fischer & Karl, 2019) Thus, observed differences in the analyzed indicators were negligible. Specifically, chi-square differences between the constrained and unconstrained models were not statistically significant (i.e., Δχ^2^/Δdf < 1.96), and differences in CFI, TLI, and RMSEA were less than 0.01 see Table 5. These results indicate that, for the first-order four-factor model, the factor loadings, thresholds, and scalars were invariant between Brazilians and Portuguese (Fischer & Karl, 2019).

**Table 5:** Multigroup Confirmatory Factor Analysis (MCFA) by nationality (Brazil X Portugal)

Older Workers (Brazilian vs. Portuguese)	*χ*^2^	(Δ* χ*^2^)	CFI	(ΔCFI)	TLI	(ΔTLI)	RMSEA	(ΔRMSEA)
Configural	448.82	-	0.955	-	0.937	-	0.066	-
Metric	470.77	21.95	0.953	0.002	0.939	0.002	0.065	0.001
Scalar	525.84	55.07	0.947	0.006	0.936	0.003	0.067	0.002

*χ*^*2*^ chi-squared, Δ χ^2^ difference of chi-square, *CFI* Comparative Fit Index, Δ *CFI* difference of CFI, *RMSEA* Root Mean Square Error of Approximation, Δ *RMSEA* difference of RMSEA, *Configural* Factor structure fixed between groups, *Metric* Factor structure and factor loadings fixed between groups, *Scalar* Factor structure, factor loadings, thresholds and scalars fixed between groups. n Brazilians = 764; n Portuguese = 607.

### Mean Differences Between Brazil and Portugal

Differences between Brazil and Portugal regarding retirement decisions were examined using the Mann–Whitney *U* test. The results indicated statistically significant differences in three of the four latent dimensions. Participants from Portugal showed significantly higher mean scores for Early retirement (*U* = 153,761, *p* <.001; *M*Portugal = 3.08, *SD* = 1.06; *M*Brazil = 2.48, *SD* = 0.99; *r* = −.34) and Full retirement (*U* = 150,167.00, *p* <.001; *M*Portugal = 2.89, *SD* = 1.08; *M*Brazil = 2.25, *SD* = 0.96; *r* = −.35), both with moderate effect sizes, indicating a greater tendency toward retirement among Portuguese participants.

In contrast, participants from Brazil reported significantly higher mean scores for Bridge employment (*U* = 288,550.00, *p* <.001; *M*Brazil = 3.19, *SD* = 0.85; *M*Portugal = 2.81, *SD* = 0.94), with a small effect size (*r* =.24), suggesting a greater inclination toward continuing to work. No statistically significant difference was observed between Brazil and Portugal for Late retirement (*U* = 242,742.00, *p* =.13; *M*Brazil = 2.21, *SD* = 0.83; *M*Portugal = 2.13, *SD* = 0.85; r =.05). Overall, the findings indicate that participants in Portugal tend to favor retirement decisions, whereas participants in Brazil tend to favor continued labor market participation.

The final 13-item RDI, with items grouped by subscale, is presented in Table 6.

**Table 6:** Item Version of the Retirement Decision Inventory (RDI) with Subscales

Dimension	Items
Early retirement	RDI1—I would like to retire before the legal retirement period (age + length of service)
	RDI3—I would like to stop working as soon as possible
	RDI4—I don''t see myself working to retirement age or length of service
	RDI5—I need to retire before the legal retirement period
Full retirement	RDI7—When I retire, I won''t work at anything else
	RDI8—For me, retirement and work don''t go very well together
	RDI9—I want to retire and feel free of all work
Bridge employment	RDI12—After being granted statutory retirement, I would like to have a job in another company, at reduced hours
	RDI13—After being granted statutory retirement, I would like to work on a different project for a fixed duration
	RDI14—After being granted statutory retirement, I intend to work in other activities a few days a week
Late retirement	RDI17—I''m going to retire and work full-time for another company
	RDI18—I''m going to retire and work for myself, full time
	RDI19—I''m going to retire and work something else, but full-time

## Discussion

This study aimed to develop and present evidence of the psychometric validity of the RDI in Brazil and Portugal. The items were initially developed from a compilation of the literature review by Amorim et al. (2022), complemented by the evaluation of judges and discussions with distinguished expert professionals in the field, resulting in a structure of 20 items distributed in four dimensions: early retirement, full retirement, bridge employment, and late retirement. This study achieved its objective by proposing an instrument capable of capturing the intensity of the different retirement intentions, as well as presenting its factor structure, internal consistency, and invariance.

The creation of the RDI aims to fill existing gaps in the measurement of workers'' intentions when faced with the various possibilities at the time of retirement. This instrument not only makes it easier for older workers to reflect on their options but is also a valuable tool for human specialists and professionals in health, organizational, and developmental psychology.

The exploratory and confirmatory analyses carried out with 1,371 participants (764 Brazilians and 607 Portuguese) indicated that the fit indices of the RDI model were adequate, with satisfactory to good internal consistency (Cronbach''s α >.70; McDonald''s ω >.70) and satisfactory factor loadings (>.50). The final version of the RDI consists of 13 items grouped into four dimensions, with configural, metric, and scalar invariance observed between Brazil and Portugal. These results demonstrate that the instrument maintains its psychometric structure across both national contexts and can be used by organizations that offer retirement transition planning for older workers**.**

The observed differences between Brazilian and Portuguese participants may also be interpreted through culturally embedded orientations toward aging, work, and social roles. Cross-cultural gerontological perspectives emphasize that retirement is not simply an institutional transition but a socially and culturally constructed process in which meanings attributed to work, leisure, family responsibilities, and later life may differ across contexts (Luborsky & LeBlanc, 2003). In this regard, the higher Portuguese scores for early and full retirement may be consistent with a social representation of retirement that places greater value on disengagement from formal employment and the reorganization of daily life around leisure and family roles.

Previous research in Portugal has described retirement as a period associated with greater peace, leisure, and opportunities to support family members, particularly descendants (Fonseca, 2012; Loureiro et al., 2022). In contrast, the higher Brazilian scores for bridge employment may reflect, at least in part, a normative orientation in which continued economic and productive participation remains closely connected to family responsibilities and social roles in later life. Brazilian research has shown that continued work after retirement may be experienced not only as an economic necessity but also as a meaningful form of continued participation (Andrade & Torres, 2020; França et al., 2013; Guerson et al., 2018).

Some human resources professionals and academics have found it difficult to understand what bridge employment really means, particularly for older workers in different national contexts. The bridge employment dimension of the RDI warrants particular attention, as this concept may be less familiar or more ambiguous in certain professional and cultural contexts.

This may occur because some professions, practiced for many years in highly specialized areas or subject to strict institutional regulations, may limit workers'' ability to envision continuity models beyond complete withdrawal from active professional life (Luborsky & LeBlanc, 2003). To resolve the ambiguities identified during the validation process, we clarified that bridge employment in this inventory specifically refers to individuals who have statutory retirement rights yet choose to continue working in flexible arrangements (Beehr & Bennett, 2015).

Research suggests that adopting bridge employment and supporting the sustainable employability of workers who wish to continue contributing to society benefits countries'' economies (Leinonen et al., 2020), healthcare systems (Frimmel & Pruckner, 2020), and the life satisfaction of retirees who return to the labor market, as observed among Brazilian participants (Guerson et al., 2018). However, bridge employment remains relatively uncommon in European countries such as Portugal, where recently retired individuals often prioritize rest and leisure activities, as well as opportunities to support their descendants'' families (Loureiro et al., 2022).

Furthermore, the statutory retirement age in Portugal is higher than in Brazil, which could potentially contribute to differences in functional capacity and work readiness at the point of retirement eligibility, though this relationship warrants further investigation.

On the other hand, even though older people may wish to continue working, post-retirement employment is not a widely adopted practice in Portuguese society, largely because ageist stigma persists in contemporary European societies (Stecker et al., 2025).

It should be noted that even if older workers choose to retire at the statutory age, this decision does not necessarily preclude future labor market participation. Returning to work depends on both the worker''s desire and/or need to do so, as well as the availability of bridge employment opportunities. Given the cross-national differences in bridge employment practices and the conceptual ambiguities identified during instrument development, we recommend revising the three bridge employment items to enhance clarity and cultural appropriateness. The proposed revisions explicitly reference statutory retirement and emphasize the diverse forms that post-retirement work can take: Item 12—After being granted statutory retirement, I would like to have a job in another company at reduced hours; Item 13—After being granted statutory retirement, I would like to work on a different project for a fixed duration; and Item 14—After being granted statutory retirement, I intend to work in other activities a few days a week.

### RDI Limitations

Despite the contributions of this study, it is necessary to highlight some limitations that should be considered when interpreting the results. One of the main limitations is related to the characteristics of the Brazilian sample, composed predominantly of individuals with a high level of education (undergraduate and postgraduate), high income, and concentrated mainly in the Southeast region. These aspects do not reflect the diversity of the Brazilian population, which may compromise the generalizability of the results to other regions and socioeconomic groups. Brazil, being a country with a large population and area, requires substantial financial resources to conduct a representative analysis that would be ideal. In the case of this sample, it is noted during data interpretation that results should not be generalized, especially Brazil due to the education level of the sample.

Educational attainment is closely associated with greater access to information, higher perceived control, and more opportunities for planning and decision-making throughout the life course. Consequently, participants with higher education may be more inclined to perceive retirement options as feasible and desirable pathways, potentially amplifying continuity-oriented decision patterns. This sample characteristic may therefore influence the relative salience of specific retirement decision options rather than the underlying structure of the construct itself. This pattern may reflect the practical challenges of recruiting a socioeconomically representative sample in a country as large and populous as Brazil, which typically requires substantial resources. Future research should replicate these findings in samples that are more socioeconomically diverse and representative (Instituto Nacional de Estatística [INE], 2024); Instituto Brasileiro de Geografia e Estatística [IBGE], 2023).

A further limitation relates to the RMSEA value observed in the confirmatory factor analysis, which remained above the conventional cutoff adopted in this study (RMSEA =.097, 90% CI [.088,.106]). Although the CFI (.969) and TLI (.959) indicated favorable model fit and the standardized factor loadings were consistently high, the elevated RMSEA suggests that the fit of the model should be interpreted with caution. As RMSEA may be sensitive to model characteristics, including the number of observed variables, this result does not necessarily indicate inadequate model specification when considered alongside the remaining fit indices. Nevertheless, replication of the RDI factor structure in independent and more diverse samples is recommended to further examine the stability and adequacy of the model. Another limitation concerns the cross-sectional nature of the data. This design restricts the ability to examine how retirement intentions evolve and translate into concrete actions; longitudinal studies are needed to better capture this dynamic.

Beyond addressing these limitations, the validated RDI has important practical implications for multiple stakeholders involved in retirement planning and workforce management.

### Recommendations

Given that the present study provides initial evidence of validity for the RDI, the following recommendations should be regarded as provisional and subject to further validation across different populations, occupational contexts, and longitudinal designs. The RDI may have potential applications for workers, organizations, and policymakers, provided that its results are interpreted as indicators of retirement intentions rather than as definitive predictions of future behavior.

1. For workers, the RDI may be used as a self-reflection tool during retirement planning. It may help older workers reflect on their intentions and expectations and consider different possibilities across the four dimensions, including formal or informal work, temporary or fixed-term contracts, reduced working hours, service provision, consultancy, self-employment, or entrepreneurship.
2. Retirement should not necessarily be understood as permanently preventing future labor-market participation. The RDI may help identify intentions regarding flexible or full-time post-retirement work, although actual return to employment is also likely to depend on education and skills updating, physical and cognitive conditions, individual circumstances, and the availability of appropriate employment opportunities.
3. In organizational settings, the RDI may potentially assist Human Resources professionals in understanding workers'' retirement intentions and in informing discussions about selection, training, skills updating, and possible extended employment. However, given the current stage of validation, its use should remain exploratory and should not be used to disadvantage workers or make employment decisions without additional evidence. Any organizational application should follow principles of autonomy, privacy, confidentiality, and employee well-being.
4. The information obtained through the RDI may potentially contribute to the development of strategies involving knowledge transfer, mentoring, career planning, and retirement preparation. Further research is needed to determine whether RDI-informed interventions improve retirement planning or transition outcomes.
5. Any future application of the RDI should prioritize ethical and secure data collection, including anonymity, confidentiality, and appropriate data protection procedures. These safeguards are particularly important when the instrument is used in organizational or employment-related contexts.
6. At the organizational level, RDI results may potentially contribute to discussions about financial education, retirement planning, and age-management practices. However, the effectiveness of such applications should be examined empirically before the RDI is incorporated into formal organizational programs.
7. Organizations may also consider whether flexible work arrangements and other age-management practices could support older workers who wish to remain employed. The RDI may provide one source of information for such discussions, but further research is needed to establish how retirement intentions relate to organizational retention practices and employment outcomes.
8. For governments in Brazil and Portugal, aggregated RDI findings may potentially contribute to discussions about active aging, retirement planning, and policies supporting older workers'' labor-market participation. Future research should examine whether RDI data can complement existing administrative and population-level indicators and whether its use can meaningfully inform the evaluation or development of retirement policies.

## Concluding Remarks

The psychometric validity evidence presented in this study demonstrates that the RDI is a useful tool for older workers, organizations, and governments in planning the transition to retirement. By measuring the intentions of older workers when faced with available retirement options, early retirement, full retirement, bridge employment, and late retirement, the RDI contributes to informed retirement decision-making. The instrument demonstrated adequate to good psychometric properties and supported measurement invariance across Brazilian and Portuguese contexts. Future research should broaden understanding of the RDI''s applicability across different contexts and populations. Longitudinal studies and interventions based on the RDI could contribute to the development of more targeted support programs for older workers navigating retirement transitions.

## Data Availability

The data used in this study were collected from a survey of 1.371 Brazilian and Portuguese participants aged 50 and over, comprising sociodemographic information and responses to inventory (RDI- Retirement Decision Inventory). Given the sensitive nature of the data, which includes personal and occupational information from a specific and identifiable group of professionals, public disclosure could inadvertently compromise participant confidentiality. Additionally, public access to such detailed occupational and sociodemographic data could lead to unintended consequences, such as stigmatization or misuse of information. To uphold ethical research standards and comply with the guidelines established by the approving ethics committee, the full dataset cannot be made publicly available. However, anonymized and partial datasets, limited to variables relevant for scientific validation, may be shared upon reasonable request. Requests will be evaluated on a case-by-case basis and must comply with all ethical and legal requirements. Interested researchers may contact the corresponding author at lucia.franca@gmail.com for further inquiries.
